# Associations between self-reported sleep characteristics and incident mild cognitive impairment: The Heinz Nixdorf Recall Cohort Study

**DOI:** 10.1038/s41598-020-63511-9

**Published:** 2020-04-16

**Authors:** Christian Brachem, Angela Winkler, Sarah Tebrügge, Christian Weimar, Raimund Erbel, Karl-Heinz Jöckel, Andreas Stang, Nico Dragano, Susanne Moebus, Bernd Kowall, Martha Jokisch

**Affiliations:** 1 Nutritional Epidemiology, Department of Nutrition and Food Science, Rheinische Friedrich-Wilhelms-University Bonn, Bonn, Germany; 2 Department of Neurology, University Hospital of Essen, University of Duisburg-Essen, Essen, Germany; 3 Institute for Medical Informatics, Biometry and Epidemiology, University Hospital Essen, University Duisburg-Essen, Essen, Germany; 4 Center of Clinical Epidemiology, Institute for Medical Informatics, Biometry and Epidemiology, Medical Faculty, University Duisburg-Essen, Essen, Germany; 5 School of Public Health, Department of Epidemiology Boston University, 715 Albany Street, Talbot Building, Boston, MA 02118 USA; 6 Institute of Medical Sociology, Medical Faculty, University of Duesseldorf, Duesseldorf, Germany

**Keywords:** Epidemiology, Sleep disorders

## Abstract

Associations of sleep characteristics with mild cognitive impairment (MCI) have been examined in cross-sectional, but rarely in longitudinal studies. Incident MCI and sleep characteristics were assessed in 1,890 participants of the first and second follow-up of the Heinz Nixdorf Recall study, a population-based cohort study in Germany (age at first follow-up 50−80 years, mean follow-up 5.2 years). MCI was assessed with extensive cognitive tests. Sleep questionnaires including PSQI (Pittsburgh Sleep Quality Index) were used to assess sleep quality, sleep disturbances, time asleep, and time in bed. Relative risks (RR) of developing MCI when exposed to sleep characteristics were assessed in regression models adjusted for sociodemographic and cardiovascular risk factors. Poor sleep quality (PSQI > 5) (RR = 1.43, 95% CI: 1.12−1.82, fully adjusted, reference: PSQI ≤ 5) and difficulties initiating sleep (almost nightly versus never) (RR = 1.40, 0.94−2.08) were associated with incident MCI. For time in bed, the risk of MCI was increased for ≤ 5 hours (RR = 2.86, 1.24─6.60, reference:7 to <8 hours). In this longitudinal study with older participants, MCI risk was increased in persons with poor sleep quality, difficulties initiating sleep, and short time in bed.

## Introduction

Dementia is a growing public health burden worldwide. In 2013 an estimated 44.35 million persons had a prevalent dementia^1^. By 2050 it is expected that this number triples to 135.46 million prevalent dementia cases^2,3^. Because no effective causal medical therapies are available for dementia, primary prevention of dementia and of its early precursors is the most promising option currently available to cap the rising prevalence^4,5^.

Subjects with mild cognitive impairment (MCI) have an increased risk of progression to Alzheimer’s disease (AD) and other forms of dementia. Therefore, identification of modifiable risk factors for incident MCI is important, and poor sleep is considered a potential risk factor for cognitive decline and disease progression^6–13^.

Sleep characteristics were suggested as modifiable risk factors for cognitive decline, for example by influencing hippocampal volume^14,15^. Two up-to-date reviews on this relationship indicate an association between cognitive decline and sleep problems, such as poor sleep quality, short or long sleep duration, and sleep disturbances^12,16^. However, both reviews concluded that there is still a need for long term prospective studies to ensure that sleep problems precede cognitive decline^12,16^. Two recently published cohort studies on sleep characteristics and dementia were not included in these reviews^17,18^. Both suggest that self-reported long sleep duration as well as self-reported sleep disturbances increase the risk of dementia. Additionally, Jackowska and Cadar^19^ found an association between decreased cognitive function and self-reported long and short sleep duration in their prospective cohort study. However, there is still a knowledge gap for longitudinal studies on sleep characteristics and mild cognitive impairment.

Using longitudinal data of the Heinz Nixdorf Recall Study (HNR Study), this study aims to fill this knowledge gap and to extend a previous cross-sectional analysis of HNR Study data^11^. To this purpose, we examined the relationship between multiple sleep exposures (overall sleep quality; difficulties maintaining or initiating sleep, early-morning awakening; time in bed, time asleep (i.e. sleep duration without time awake in bed) and total sleep duration) and incidence of MCI.

## Methods

### Participants

The HNR Study is a population-based prospective cohort study conducted in three large adjacent cities (Bochum, Essen, Mülheim) in the Ruhr-region of North-Rhine-Westphalia, Germany. The study rationale and design have been described in detail elsewhere^20,21^. In short, the cohort comprises a total of 4,814 participants (49.8% men, aged 45–75 years). The baseline visits (t0) were performed between 2000 and 2003. The median follow-up time was 5.1 years for the 5-year follow-up visits between 2005 and 2008 (t1), and 5.2 years for the 5-year follow-up visits between 2010 and 2015 (t2). Data assessment at baseline and at follow-up visits included a self-administered questionnaire, face-to-face interviews, and a physical examination including among others anthropometric measurements and comprehensive laboratory tests. Permission to conduct this study was granted by the Institutional Review Board (IRB) of the Medical Faculty of the University of Duisburg-Essen (Approval Number: 99-69-1200). The study was performed in accordance with the approved guidelines and regulations. All participants gave their written informed consent.

**Figure 1:**
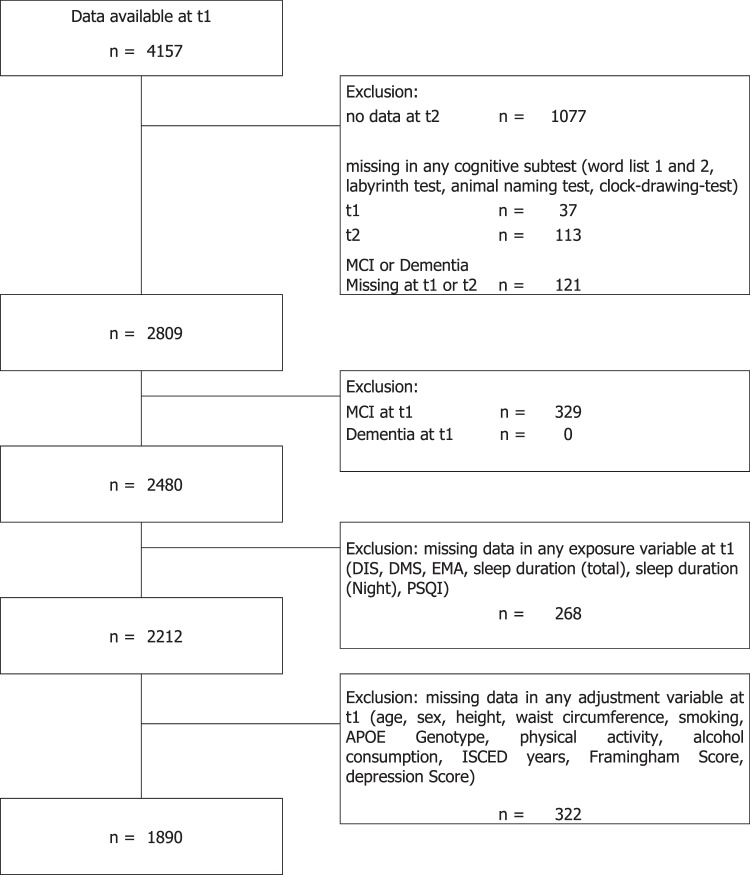
Flow-chart of persons entering the complete case analysis dataset. Abbreviations: MCI - Mild Cognitive Impairment; Cont. - Continuous Cognition Variables; DIS - Difficulties Initiating Sleep; DMS - Difficulties Maintaining Sleep; EMA - Early-morning Awakening; PSQI - Pittsburg Sleep Quality Index; ISCED - International Standard Classification of Education.

In the HNR Study, cognitive tests were performed at the second (t1) and the third visit (t2) at the study centre. Participants with a diagnosis of dementia by a physician or taking cholinesterase inhibitors (anatomic-therapeutic-chemical classification issued by the World Health Organization (ATC) code: N06DA)) or other anti-dementia drugs (ATC: N06DX) or fulfilling the DSM-IV dementia diagnosis at t1, were excluded from the regression analysis. As our outcome of interest was incident MCI at t2, participants with MCI at t1 were excluded as well. Participants were included into the present study if data were available for all variables of interest for the complete case analysis (Fig. 1; n = 1890).

### Cognitive assessment

The cognitive performance at t2 was measured using eight neuropsychological subtests: (1) immediate and (2) delayed recall of eight words (performance measured as number of words recalled in each trial)^22^, (3) labyrinth test (time in seconds needed to find the way out of a maze)^22^, (4) verbal fluency “animals” (number of words named within one minute)^23^, (5) clock-drawing test (performance was rated from 1 (perfect clock) to 6 (poor performance))^24^, (6,7) Trail Making Test A and B (A: time in seconds needed to connect a series of consecutively numbered circles; B: time in seconds needed to connect a series of numbered and lettered circles in alternating sequence)^25^, (8) colour-word test^22^ (time in seconds needed to read written colour words inked in black (card 1), time in seconds needed to name coloured bars (card 2), time in second needed to name the colour of written colour words, when the written word and printed ink was not congruent (card 3) and difference card 3 – card 2 as interference performance). Tests were grouped into four domains (see Table 1). The first five subtests had already been performed at t1. For a detailed description of tests (1) to (5) see Wege *et al*. (2011)^26^ and of tests (6) to (8) see Tebrügge *et al*. (2018)^27^. For the five subtests that have already been performed at t1, we performed z-transformation of the raw data at t2 using our own defined norm-data from t1: raw data were z-transformed based on the mean and standard deviation (SD) of the appropriate age- and education group at t1 (age: 50 to 59 years, 60 to 69 years, and ≥ 70 years; education: ≤10 years, 11 to 13 years, and ≥ 14 years)^28^. For the three subtests that were additionally conducted at t2, z-transformation was based on the same education groups and the following three age groups from t2: 55 to 64 years, 65 to 74 years and ≥ 75 years^28^. Except for the clock-drawing test, the age- and education-adjusted test scores were scaled to have a mean of 10 and a SD of 3^28^. Within each domain (attention, executive function, verbal memory and visuoconstruction), newly scaled scores of the tests were added. To account for the differing numbers of tests in each domain, domain scores were then scaled to have a mean of 10 and a SD of 3^28^. Cognitive impairment was defined as a performance of more than one SD below the mean (≤7) in at least one total domain score of the domains attention, executive function, verbal memory, or as a score of ≥ 3 in visuoconstruction^28^.

**Table 1:** Description of cognitive domains.

Cognitive Domain	Tests
Attention	Trail Making Test A, Color-word test card 1 and card 2
Executive Function	Trail Making Test B, Labyrinth test, Color-word test interference performance (card 3 – card 2)
Verbal Memory	Eight-word list immediate and delayed recall
Visuoconstruction	Clock-drawing test

### Definition of mild cognitive impairment

The MCI diagnosis at t2 was based on the following published MCI criteria^29,30^: (1) cognitive impairment in at least one of the above reported four domains; (2) subjective cognitive decline over the past two years; (3) generally intact activities of daily living; (4) no dementia diagnosis^28^. To examine incident MCI, participants with MCI at t1 were excluded. Because cognitive assessment at t1 consisted of only five subtests, we were unable to build cognitive domain scores at this time-point. Thus, the first MCI criterion (cognitive impairment) was met if the participant performed more than one SD below the age and education-adjusted mean in at least one subtest or had a score of ≥ 3 in the clock-drawing test. Participants at t2 not meeting MCI criteria as described above were categorized as ‘no MCI’^28^.

### Assessment of sleep characteristics

Sleep variables were assessed in a computer-assisted interview. In addition to the Pittsburgh Sleep Quality Index (PSQI)^31^, we asked six questions, three on sleep disturbances, and three on sleep duration.

### Sleep quality: PSQI

The PSQI is a standardised, self-rated questionnaire that measures sleep quality. The questionnaire consists of seven sub-scores that are described in detail elsewhere^31^. Poor overall sleep quality is indicated by a PSQI score of >5^31^, and accordingly, PSQI was grouped to scores of ≤ 5 and> 5 in the regression analyses.

### Sleep disturbances

The additional questions about sleep disturbances were: “Have you experienced difficulties initiating sleep during the past four weeks?”, “Have you experienced difficulties maintaining sleep during the past four weeks?” and “Have you experienced early-morning awakening during the past four weeks?” Each question had the following answer possibilities: never (during the past four weeks), sometimes (once or less than once a week), often (at least twice a week), and almost every night (at least five nights a week). The reference category for regression analysis was “*never (during the past four weeks)”*.

### Nocturnal sleep duration

We used the two following variables to assess self-reported nocturnal sleep duration:

1. “Time asleep” (i.e., sleep duration without time awake in bed) which is assessed using an item from the PSQI (“During the past month, how many hours of actual sleep did you get at night? This may be different than the number of hours you spend in bed.”).
2. “Time in bed” was calculated from two items in the PSQI (“During the past month, when have you usually gone to bed?”, “During the past month, when have you usually got up in the morning?”).

### Duration of napping and total sleep duration

In addition, we asked two questions on frequency and duration of napping (“How frequently do you usually nap?”, and “How many hours do you usually nap?” assessed as hours and minutes). The question about nap frequency had the following choices: never, less than once a week, one to four times per week, five to six times per week, and daily. Mean duration of daytime napping was calculated as reported duration of daytime napping multiplied by its relative frequency. Total sleep duration was calculated as the sum of time in bed during the night plus duration of daytime napping.

Time asleep, time in bed, and total sleep duration were categorised according to the American National Sleep Foundation’s recommendations^32^ into: ≤ 5 hours,> 5 to <7 hours, 7 to <8 hours (reference), 8 to <9 hours and ≥ 9 hours. Sleep duration was categorised because of the hypothesized non-linear dependency and the tendency of participants to answer in half-hour intervals.

### Covariates

As part of the first follow-up visit of the HNR Study at t1, information about medical and family history, cardiovascular risk factors, lifestyle and various sociodemographic factors were assessed in a computer assisted interview^20,21^. The variables used for model adjustment were selected based on a directed acyclic graph (DAG) based on current literature on the topic. The DAG (appendix 1) was constructed using the online software DAGity v2.3^33^. The minimally sufficient adjustment set was: age, sex, height, waist circumference, cardio-vascular disease (CVD) risk, physical activity, smoking status, International Standard Classification of Education (ISCED) years, pure alcohol consumption, Apolipoprotein E (APOE) ε4 genotype, and depression score. Age (in years), sex (male, female), height (cm), waist circumference (cm), CVD risk (as Framingham Risk Score), physical activity (as Metabolic Equivalent of Tasks – hours (MET-h)/week), ISCED years, pure alcohol consumption (grams/week), and depression score (short form of the Center for Epidemiologic Studies Depression Scale, German version^34^) were used as continuous variables. Smoking status (current, former, never (reference)) and APOE ε4 genotype (ε4 carrier: at least one ɛ4 allele (ɛ2/ɛ4, ɛ3/ɛ4, ɛ4/ɛ4), non-carrier(reference)) were used as categorical variables. Further details on the covariate assessment have been described elsewhere^21,26^. We did not categorise the continuous variables used in the adjustment set.

### Statistical analysis

We used a log-linear model with a Poisson working likelihood and robust standard errors to obtain relative risks and corresponding 95% confidence intervals^35,36^. The outcome variable was incident MCI, exposure variables were global sleep quality assessed by the PSQI score, sleep disturbances, time asleep, time in bed, and total sleep duration.

For each exposure of interest, we calculated three models: first, a crude model (cf. appendix 5) with only the exposure as an independent variable; second, a model additionally adjusted for age and sex (cf. appendix 5); third, a fully adjusted model including all variables suggested by the DAG.

We performed regression analyses with complete case analyses (N = 1890), and, in addition, with multiple imputations (N = 2480) (cf. Fig. 1). Missing data was imputed with PROC MI in SAS 9.4. All variables in the fully adjusted regression model were used in the imputation model (n_impute_ = 10). A table detailing patterns of missing in all variables used in the imputation can be found in the supplementary material (cf. appendix 2–4). The overall fraction of missing data was 2.4%. This ranged from no missing data (e.g. in age and sex) to 10.6% in the PSQI score.

All statistical analysis was performed using SAS 9.4. We calculated estimates and corresponding 95% confidence intervals to assess the precision of our estimates because estimation, not significance testing, was our goal. Further, we wished to avoid publication bias by preferential reporting of statistically significant results. Instead, we judged our estimates by their precision and validity.

### Additional analyses

We used changes of sleep duration between T1 and T0 as a further exposure of interest. Changes of sleep duration (sleep duration at T1 minus sleep duration at baseline) were grouped into three categories (≤ -1 hour, -1 hour to 1 hour (reference), ≥1 hour). As the PSQI was not administered to participants at baseline, we used another item for calculation of change of sleep duration between T1 and T0: Participants were asked “How many hours do you sleep per night on average?”, and they were instructed to indicate hours and minutes in a computer assisted interview. Additionally, we stratified our cohort into three age groups (45 to 54, 55 to 64 and 65 to 74 years) to look for effect modification by age. Furthermore, we stratified our cohort by APOE carrier status to investigate effect modification. For these additional analyses, we used the same modelling procedures and adjustment sets as described above.

## Results

### Social and clinical characteristics

The sociodemographic and clinical characteristics of all 1890 participants included in the complete case analysis are presented in Table 2. Participants with short time asleep (≤ 5 hours) were more often women and had higher proportions of sleep disturbances and poor sleep quality (PSQI > 5). These participants had more difficulties maintaining sleep, more difficulties initiating sleep, and more often reported early-morning awakening. Participants with long sleep duration (≥9 hours) were the oldest, had the highest Framingham scores and the lowest education years. Alcohol consumption and depression scores were highest and physical activity the lowest in persons with 5 or less hours of nocturnal sleep duration.

**Table 2:** Baseline characteristics stratified by nocturnal sleep duration: the Heinz Nixdorf Recall Study.

Variable		Time asleep				
		≤ 5 h	> 5 h to < 7 h	7 h to < 8 h	8 h to < 9 h	≥ 9 h
N		209 (100.0%)	612 (100.0%)	655 (100.0%)	352 (100.0%)	62 (100.0%)
Sex	Female	138 (66.0%)	311 (50.8%)	336 (51.3%)	162 (46.0%)	26 (41.9%)
Age (years)		57.5 ± 7.2	56.7 ± 7.0	57.9 ± 7.0	60.2 ± 7.1	60.8 ± 6.1
Sleep Quality: PSQI Score^a^		11.0 ± 3.4	6.6 ± 2.9	4.0 ± 2.1	3.4 ± 1.9	3.2 ± 1.9
	> 5	197 (94.3%)	369 (60.3%)	134 (20.5%)	40 (11.4%)	7 (11.3%)
Difficulties Initiating Sleep	Sometimes	45 (21.5%)	231 (37.7%)	262 (40.0%)	130 (36.9%)	21 (33.9%)
	Often	64 (30.6%)	125 (20.4%)	93 (14.2%)	28 (8.0%)	6 (9.7%)
	Almost every Night	66 (31.6%)	43 (7.0%)	19 (2.9%)	8 (2.3%)	2 (3.2%)
Difficulties Maintaining Sleep	Sometimes	19 (9.1%)	142 (23.2%)	177 (27.0%)	114 (32.4%)	14 (22.6%)
	Often	60 (28.7%)	164 (26.8%)	180 (27.5%)	92 (26.1%)	12 (19.4%)
	Almost every Night	124 (59.3%)	255 (41.7%)	209 (31.9%)	104 (29.5%)	29 (46.8%)
Early-morning Awakening	Sometimes	45 (21.5%)	187 (30.6%)	227 (34.7%)	117 (33.2%)	19 (30.6%)
	Often	62 (29.7%)	144 (23.5%)	98 (15.0%)	44 (12.5%)	1 (1.6%)
	Almost every Night	62 (29.7%)	76 (12.4%)	25 (3.8%)	10 (2.8%)	3 (4.8%)
APOE ε4 Genotype	ε4 Carrier^b^	42 (20.1%)	152 (24.8%)	173 (26.4%)	85 (24.1%)	15 (24.2%)
Alcohol Consumption [g/week]	9.77 ± 17.39	9.8 ± 17.4	8.1 ± 13.8	8.9 ± 15.4	9.4 ± 16.2	
Depression Score^c^		9.1 ± 5.9	7.7 ± 5.9	6.7 ± 5.4	6.6 ± 5.4	6.5 ± 6.2
Framingham Risk Score		0.2 ± 0.2	0.2 ± 0.2	0.2 ± 0.2	0.3 ± 0.2	0.3 ± 0.2
Height [cm]		167.1 ± 9.4	169.4 ± 9.0	169.5 ± 9.3	169.5 ± 9.1	170.9 ± 9.0
ISCED [years]		13.9 ± 2.1	14.5 ± 2.4	14.5 ± 2.4	14.3 ± 2.3	13.8 ± 2.1
Physical Activity [MET-h/Week]	40.64 ± 44.81	40.6 ± 44.8	42.1 ± 39.3	45.6 ± 42.2	52.4 ± 55.2	
Waist Circumference [cm]		92.3 ± 14.6	91.9 ± 12.8	92.4 ± 12.4	93.4 ± 12.3	95.7 ± 14.3
Smoking Status	Former Smoker	75 (35.9%)	240 (39.2%)	239 (36.5%)	128 (36.4%)	23 (37.1%)
	Current Smoker	42 (20.1%)	126 (20.6%)	110 (16.8%)	83 (23.6%)	13 (21.0%)

All numbers were derived from the complete case analysis dataset (N = 1890). Data is displayed as N (Col Percent) or Mean ± Standard Deviation. Reference Categories were omitted. Abbreviations: PSQI: Pittsburgh Sleep Quality Index; g: Gramm; cm: Centimetres; ISCED: International Standard Classification of Education; MET-h: Metabolic Equivalent of Task – hours, APOE. Apolipoprotein E.

^a^Score > 5 indicates poor sleep quality ^b^ɛ4 carrier: at least one 4 allele (ɛ2/ɛ4, ɛ3/ɛ4, ɛ4/ɛ4) ^c^Centre for Epidemiologic Studies Depression Scale, higher scores indicate higher levels of depression.

### Sleep quality and PSQI score

A PSQI score > 5 was associated with an increased risk for MCI (RR = 1.43, 95% CI: 1.12 to 1.82)) in the fully adjusted model with multiple imputations, see Table 3. This was confirmed when the PSQI score was used as a continuous variable (RR = 1.05 (1.01 to 1.08)).

**Table 3:** Adjusted relative risks for the association between sleep quality, and sleep disturbances, respectively, and the incidence of mild cognitive impairment at t2: the Heinz Nixdorf Recall Study.

Exposure		Complete Case Analysis (N = 1890)				Multiple Imputation (N = 2480)		
		N	n (%)	RR	95% CI	n %	RR	95% CI
Sleep Quality: PSQI Score	≤ 5 (good)	1143	105 (9.2%)	1.00	Reference	9.0%	1.00	Reference
	> 5 (poor)	747	96 (12.9%)	1.38	[1.06 to 1.80]	13.5%	1.43	[1.12 to 1.82]
Difficulties Initiating Sleep	Never	747	75 (10.0%)	1.00	Reference	10.0%	1.00	Reference
	Sometimes	689	70 (10.2%)	1.04	[0.76 to 1.42]	10.0%	1.02	[0.78 to 1.35]
	Often	316	35 (11.1%)	1.12	[0.76 to 1.64]	12.8%	1.25	[0.91 to 1.72]
	Almost every Night	138	21 (15.2%)	1.46	[0.94 to 2.28]	15.2%	1.40	[0.94 to 2.08]
Difficulties Maintaining Sleep	Never	195	20 (10.3%)	1.00	Reference	10.6%	1.00	Reference
	Sometimes	466	41 (8.8%)	0.89	[0.54 to 1.47]	9.2%	0.85	[0.56 to 1.31]
	Often	508	50 (9.8%)	0.96	[0.59 to 1.56]	10.2%	0.91	[0.60 to 1.37]
	Almost every Night	721	90 (12.5%)	1.10	[0.70 to 1.72]	12.5%	1.04	[0.71 to 1.52]
Early Morning Awakening	Never	770	79 (10.3%)	1.00	Reference	10.4%	1.00	Reference
	Sometimes	595	58 (9.7%)	0.99	[0.72 to 1.37]	10.0%	0.99	[0.75 to 1.30]
	Often	349	46 (13.2%)	1.30	[0.92 to 1.84]	13.3%	1.25	[0.93 to 1.69]
	Almost every Night	176	18 (10.2%)	0.99	[0.61 to 1.60]	10.9%	0.99	[0.66 to 1.50]

Estimates of relative risks with 95% confidence intervals were obtained from a log-linear model with a Poisson working likelihood and robust standard errors.

Abbreviations: t1: second visit in the Heinz Nixdorf Recall Study (HNR Study); t2: third visit in the HNR Study; RR: relative risk; n %: percent of cases; CI: Confidence Interval.

Model is adjusted for age (cont.) and sex (male/female), height (cont.), waist (cont.), Framingham CVD risk score (cont.), smoking status (never/former/current), ISC education years (cont.), physical activity (cont.; MET-h/week), pure alcohol consumption (cont.; g/week), APOE e4 genotype (carrier/non-carrier), and depression score (cont.).

#### Sleep disturbances

After multiple imputation, the relative risk for MCI was increased for persons experiencing difficulties initiating sleep almost every night (RR = 1.40 (0.94 to 2.08)), but not for experiencing difficulties maintaining sleep and early morning awakening, respectively (cf. Table 3).

#### Sleep duration

**Figure 2:**
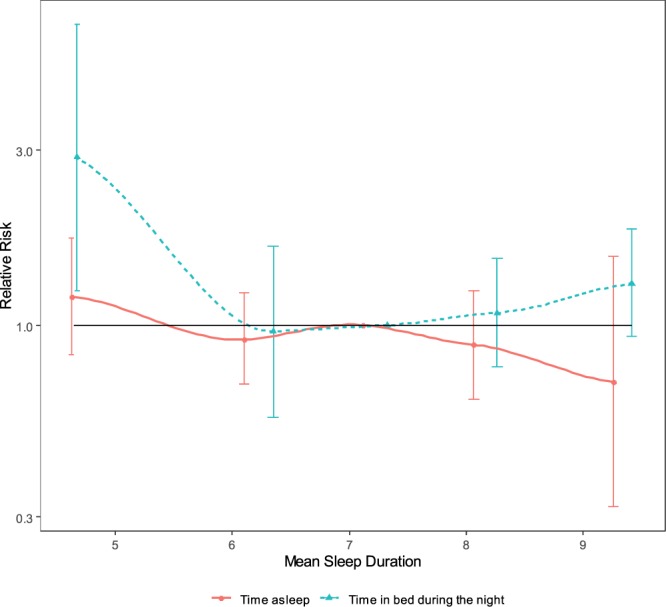
Relative Risks for Mild Cognitive Impairment: The Heinz Nixdorf Recall Study. Estimates of relative risk were obtained from log-linear model with a Poisson working likelihood and robust standard errors. Missing values were imputed. Sleep duration was assesed as a categorical variable. Dots are the means of their respective category. Categories were: ≤ 5h | > 5 to < 7h | 7 to < 8h (reference) | 8 to < 9h | ≥ 9h.

We observed a U-shaped association between time in bed during the night and incident MCI as well as between total sleep duration (time in bed during the night plus daytime napping) and incident MCI (cf. Table 4, cf. Fig. 2). For nocturnal time in bed, persons who spent ≤ 5 hours in bed, and persons who spent ≥ 9 hours in bed were more likely to develop MCI than persons who spent 7 to <8 hours in bed (RR = 2.86 (95% CI: 1.24 to 6.60), and RR = 1.30 (95% CI: 0.93 to 1.82), respectively). For time asleep (which does not include time awake in bed) results were different: The RR for incident MCI was 1.19 (95% CI: 0.83 to 1.72) for persons with ≤ 5 hours of time asleep, and it was 0.70 (0.32 to 1.54) for persons with ≥ 9 hours.

**Table 4:** Adjusted relative risks for the association between subjective sleep duration and the incidence of mild cognitive impairment at t2: the Heinz Nixdorf Recall Study.

Exposure		Complete Case Analysis (N = 1890)				Multiple Imputation (N = 2480)		
		N	n (%)	RR	95% CI	n %	RR	95% CI
Time in bed during the night^a^	≤ 5 h	13	3 (23.1%)	3.14	[1.16 to 8.49]	22.8%	2.86	[1.24 to 6.60]
	> 5 h to <7 h	209	13 (6.2%)	0.82	[0.45 to 1.50]	8.1%	0.96	[0.56 to 1.64]
	7 h to <8 h	472	40 (8.5%)	1.00	Reference	9.0%	1.00	Reference
	8 h to <9 h	655	70 (10.7%)	1.09	[0.75 to 1.59]	10.7%	1.08	[0.77 to 1.52]
	≥ 9 h	535	75 (14.0%)	1.33	[0.92 to 1.94]	13.4%	1.30	[0.93 to 1.82]
Time asleep^b^	≤ 5 h	209	28 (13.4%)	1.28	[0.86 to 1.90]	13.5%	1.19	[0.83 to 1.72]
	> 5 h to <7 h	612	58 (9.5%)	0.91	[0.65 to 1.26]	10.0%	0.91	[0.69 to 1.22]
	7 h to <8 h	655	71 (10.8%)	1.00	Reference	11.2%	1.00	Reference
	8 h to <9 h	352	39 (11.1%)	0.90	[0.62 to 1.30]	10.7%	0.88	[0.63 to 1.24]
	≥ 9 h	62	5 (8.1%)	0.66	[0.28 to 1.59]	8.5%	0.70	[0.32 to 1.54]
Total Sleep Duration^a,c^	≤ 5 h	7	2 (28.6%)	2.01	[1.27 to 3.19]	21.8%	2.63	[0.70 to 9.85]
	> 5 h to <7 h	198	13 (6.6%)	1.09	[0.75 to 1.58]	8.9%	1.11	[0.65 to 1.92]
	7 h to <8 h	447	33 (7.4%)	1.00	Reference	8.4%	1.00	Reference
	8 h to <9 h	627	71 (11.3%)	1.35	[0.95 to 1.92]	11.1%	1.19	[0.83 to 1.70]
	≥ 9 h	605	82 (13.6%)	1.65	[1.01 to 2.68]	13.0%	1.31	[0.92 to 1.85]

Estimates of relative risks with 95% confidence intervals were obtained from a log-linear model with a Poisson working likelihood and robust standard errors.

Abbreviations: t1: second visit in the Heinz Nixdorf Recall Study (HNR Study); t2: third visit in the HNR Study; RR: relative risk; n %: percent of cases; CI: Confidence Interval

Model is adjusted for age (cont.) and sex (male/female), height (cont.), waist (cont.), Framingham CVD risk score (cont.), smoking status (never/former/current), ISC education years (cont.), physical activity (cont.; MET-h/week), pure alcohol consumption (cont.; g/week), APOE e4 genotype (carrier/non-carrier), and depression score (cont.).

^a^Complete Case N = 1884, because of 6 additional missing data for time in bed.

^b^Time asleep does not include time awake in bed.

^c^Total sleep duration includes time in bed during the night plus daytime napping.

#### Multiple imputations

The use of multiple imputations (MI) yielded different results compared to the complete case (CC) analysis. MI resulted in more precise estimations. The 95% CIs in the MI analysis were on average 13% smaller. Point estimates for some of the relative risks diverged.

#### Additional analyses

We calculated change of sleep duration between T1 and T0 from sleep duration measured at T1 and T0. Persons who reported shorter nocturnal or total sleep duration at T1 compared to T0 had a higher risk of MCI: a decrease of sleep duration of at least one hour was associated with a 1.23 (1.00 to 1.51) and 1.26 (1.02 to 1.55) fold MCI risk for change of nocturnal and total sleep duration, respectively (cf. Table 5). In age-stratified analyses, the U-shaped association between time in bed and total sleep duration and incident MCI was strongly pronounced in the two older age groups (55 to 64, 65 to 74 years), but not in the youngest. However, in the youngest group (45 to 54 years), an increased MCI risk was not observed for any sleep duration parameter, but more strongly for sleep quality parameters (cf. appendix 6). In the analysis stratified by the APOE carrier status, we observed a stronger influence of difficulties maintaining sleep, early morning awakening and sleep quality in non-carriers, e.g. for PSQI Score> 5, the RR was 1.61 (1.28 to 2.02) in non-carriers compared to 1.21 (0.82 to 1.76) in carriers. For carriers, on the other hand, short sleep duration had a larger effect on MCI risk: for ≤ 5 h of total sleep duration, the RR was 2.20 (1.22 to 3.96) in carriers compared to 1.52 (1.05 to 2.20) in non-carriers (cf. appendix 7).

**Table 5:** Relative Risks of Incident MCI for Changes in Sleep Duration Between First and Second Visit to the Study Center: the Heinz Nixdorf Recall Study^a^.

Exposure		RR	95% CI
Change of nocturnal sleep duration^b^	≤ −1h	1.23	[1.00 to 1.51]
	<−1h to < +1 h	1.00	Reference
	≥ +1 h	0.98	[0.78 to 1.23]
Change of total sleep duration^b^	≤ −1h	1.26	[1.02 to 1.55]
	<−1h to < +1 h	1.00	Reference
	≥ +1 h	0.92	[0.73 to 1.16]

Estimates of relative risks with 95% confidence intervals were obtained from a log-linear model with a Poisson working likelihood and robust standard errors. RR: Relative Risk; CI: confidence interval. Model is adjusted for age (cont.) and sex (male/female) height (cont.), waist (cont.), Framingham CVD risk score (cont.), smoking status (never/former/current), ISC education years (cont.), physical activity (cont.; meth/week), pure alcohol consumption (cont.; g/week), and depression score (cont.).

^a^Multiple imputation was used for analyses

^b^Sleep differences were calculated subtracting baseline sleeping time from T1 sleeping time.

## Discussion

In our prospective cohort study, a higher risk of MCI was observed in persons with self-reported poor overall sleep quality, in persons who reported short time in bed, and persons with a strong decrease of sleep duration before baseline. Moreover, persons who reported difficulties initiating sleep almost every night had a higher risk of incident MCI five years later. For persons reporting difficulties maintaining sleep or early morning awakening, the risk for MCI was not increased.

Results for sleep duration depended on whether time awake in bed was included or excluded in the calculation for sleep duration: For persons with short or long time asleep, there was no evidence of higher MCI risk. In contrast, the association between time in bed and incident MCI was U-shaped; however, for ≤ 5 hours of time in bed, this association was based on very few participants. The reason why relative risks for long sleep duration (≥ 9 hours) differ for time in bed and time asleep may be that persons for whom time in bed is much longer than time asleep more often have some form of sleep disturbance. This in line with the 2014 study by Blackwell *et al*. who found that self-reported long sleep durations were associated with short measured sleep duration and fragmented sleep in elderly men^37^. The differences between time in bed and time asleep for short sleep duration may be due to the fact that persons reporting ≤ 5 hours of time in bed probably suffer more from lack of sleep than persons who are actually asleep during the same time interval.

Persons with a strong decrease of sleep duration at first follow-up compared to baseline had a higher risk of MCI. This may be explained by the observation that many of these persons have become short sleepers: about 60% of persons with subjective sleep duration ≤ 5 hours at follow-up reported a decrease of sleep duration of at least one hour. However, whether change of sleep duration itself or short sleep duration is the primary cause of higher MCI risk cannot be answered by our study.

Our results based on longitudinal HNR Study data are in line with earlier cross-sectional analyses of the same cohort. Dlugaj *et al*. (2014)^11^ reported an increased Odds Ratio (OR) for MCI in participants with poor sleep quality (OR = 1.30 [1.02 to 2.03]). Poor sleep quality, however, was defined as at least one sleep disturbance reported ‘almost every night’ in contrast to a PSQI score of > 5 in the present analysis. Regarding sleep disturbances (identical item), results from the earlier cross-sectional study could be replicated in the present longitudinal study.

Similar results have also been reported in two other prospective studies on sleep patterns and cognitive decline. Potvin *et al*. (2012)^38^ found an increased OR for incidence of cognitive impairment at 12 months for men with ≤ 5 h of sleep (OR = 2.91 [1.24 to 6.82], reference> 5 to <9 h). In another prospective analysis, Keage *et al*. (2012)^39^ reported an increased relative risk for cognitive impairment when comparing ≤6.5 h to > 6.5 to <8.5 h (RR = 2.02 [1.1.7 to 3.48]) and ≥ 8.5 h to> 6.5 to <8.5 h (RR = 1.27 [0.64 to 2.48]) of nocturnal sleep. Regarding long sleep duration, several studies showed long sleep to be associated with impaired cognitive performance^13,40–42^. For instance, Ramos *et al*. (2019)^40^ found long sleep (> 9 hours) to be predictive for seven-year cognitive decline in episodic learning and memory. Potvin *et al*. (2012)^38^ reported an increased OR for women sleeping ≥ 9 h (OR = 2.10 [1.10 to 4.00], reference> 5 to <9 h). However, most studies also used self-reported sleep duration and did not explicitly exclude time awake in bed from their sleep duration calculation. In concordance, a specific pathway between long sleep duration and cognitive decline is not known. Long sleep duration is associated with cerebrovascular disease^43,44^ which is a contributor to cognitive impairment and cognitive decline^45,46^. Persons with cardiovascular disease have more often problems in maintaining sleep^47,48^. When hours awake in bed are not explicitly excluded, those persons might tend to recall longer sleep durations without subtracting time awake in bed. This might be an explanation for the vanished U-shaped association in our cohort. However, this finding has to be confirmed in studies with objective sleep measurements and incident MCI.

Our results fit well into the literature on sleep characteristics and cognitive impairment. Overall with increasing age, a dysregulation of the sleep-wake cycle resulting in more fragmented and shorter sleep is common^49^. Regarding cognitive impairment, associations with poor sleep have been found for all stages: for the preclinical stage which precedes observable cognitive impairment and which encompasses tau accumulation, beta amyloid accumulation and neurodegeneration^50^, for MCI like in the present study, and for manifest AD^16,50^. The direction of these associations, however, is hypothesized to be bi-directional^12,51,52^.

A possible mechanism for this bi-directional relationship is an interdependent cycle of brain matter damage and sleep disturbances^12,52,53^. The basal forebrain is responsible for sleep promotion in humans^54^. In MCI and AD patients, an early cell loss in this brain region was described and associated with a decline in sleep quality^55,56^. Thus, sleep problems might be a result of neurodegeneration. However, the decline in sleep quality is also associated with an increase in beta amyloid deposits (a hallmark of AD) leading to faster disease progression^51,57^. Increased beta amyloid levels in the brain impair slow-wave sleep leading to sleep problems^58,59^, thus sleep problems might be a correlate of neurodegeneration. However, even in cognitively healthy participants a shorter sleep duration and poor sleep quality was associated with beta amyloid burden^60^. In concordance, animal models showed that sleep deprivation increases interstitial fluid beta amyloid levels^61,62^. These studies indicate that poor sleep might also promote beta amyloid deposition and neurocognitive decline and impairment. This is in line with recent evidence of self-reported sleep with higher rates of hippocampal volume decline in cognitively normal participants^63^.

Overall, the association between sleep and brain health is very complex. A recent review by van Egroo *et al*. (2019)^50^ summarizes the current evidence on the hallmarks of the pathogenesis of AD and sleep. In addition to the association of sleep and beta amyloid mentioned above, animal models showed that neuronal activity as observed during extended wakefulness or chronic sleep restriction could lead to an increase in tau production^64–66^. However, it is also difficult to identify the directionality of the interplay between tau and sleep patterns in human because radiotracers for tau were not available until recently^67,68^. It is further discussed that sleep deprivation induce inflammatory responses that could adversely affect cognitive function^69^. Increased inflammatory processes also play a major role in AD^70^. Many of these factors show an interaction with beta amyloid and with the Apolipoprotein E genotype making this association even more complex^71–74^. Thus, the bi-directional pathways have to be further elucidated in studies with objective sleep measures and biomarker information throughout the life span.

Taken together, given the high prevalence of sleep problems in the elderly population and the increased prevalence of dementia cases, sleep disturbances might serve as a preclinical marker to identify individuals at risk for cognitive decline. Even when the causality is not fully understood yet, improving sleep might be a useful, reasonable and easy to administer strategy to improve brain health.

### Strengths and limitations

Strengths of our study include its population-based cohort, a large study size, its prospective design with extensive phenotyping of participants, and the in-depth measurement of cognitive abilities. Moreover, we compared different items for sleep duration, which has not been done in other studies.

A limitation of the present study is the subjective assessment of sleep patterns. However, contrary to objective measurements, self-reported sleep data do not rely on the peculiarities of a few nights in an unfamiliar environment, but rather reflect longer time periods (like, e.g., four weeks in the PSQI sleep item)^75^. Additionally, for a single night the subjective assessment from the PSQI has been shown to be comparable to polysomnography and actigraphy measurements by Zinkhan *et al*. (2014)^76^. Because this is a longitudinal study with cognitive data assessed at the first and second follow-up examination, cognitively impaired participants were less likely to participate resulting in a cohort representing a healthier population^77^. MCI was not based on clinical consensus. However, we validated the short cognitive performance assessment at t1 against a detailed neuropsychological and neurological examination in a subsample of 656 participants and observed a good accuracy in identifying MCI (area under the curve (AUC) = 0.82, 95% confidence interval (CI) = 0.78–0.85)^26^. The definition of objective cognitive impairment for the MCI diagnosis changed between t1 and t2. At t2, we were able to define cognitive domain scores for our incident MCI diagnosis as requested by Albert *et al*.^78^. Finally, we do not have any biomarker information to differentiate between underlying pathologies for incident MCI.

### Future research

A major limitation of our work is the use of subjective sleep data. For future research, objective sleep data should additionally be used. Moreover, some sleep characteristics have rarely been investigated as potential risk factors for cognitive decline like napping and sleep apnea. In earlier work, we had already investigated the association between sleep disordered breathing and MCI in a cross-sectional study where we observed a null-result [12]. For future research, a study on prevalence of sleep apnea or sleep disordered breathing and incident MCI might be a topic of interest.

## Conclusion

We found an association between various indicators for poor sleep and incident MCI. These results underline the importance of sleep for health outcomes.

## Supplementary information

Appendix.

## Data Availability

Data of the Heinz Nixdorf Recall Study can be obtained by filling a request for data use and explaining the aim of the planned analyses.
