# Supplementary material for: Associations between self-reported sleep characteristics and incident mild cognitive impairment: The Heinz Nixdorf Recall Cohort Study
Source: Sci Rep. 2020 Apr 16;10:6542. doi: 10.1038/s41598-020-63511-9 (PMC7162850; doi:10.1038/s41598-020-63511-9)
Supplement: Supplementary file 1 — Appendix. [file 41598_2020_63511_MOESM1_ESM.docx]

**Appendix: Associations between self-reported sleep characteristics and incident mild cognitive impairment: The Heinz Nixdorf Recall Cohort Study**

Christian Brachem^1^, Angela Winkler^2^, Sarah Tebrügge^2^, Christian Weimar^2^, Raimund Erbel^3^, Karl-Heinz Jöckel^3^, Andreas Stang^4,5^, Nico Dragano^6^, Susanne Moebus^3^, Bernd Kowall^4*^, Martha Jokisch^2^*

^1^ Nutritional Epidemiology, Department of Nutrition and Food Science, Rheinische

Friedrich-Wilhelms-University Bonn, Bonn, Germany.

^2^ Department of Neurology, University Hospital of Essen, University of Duisburg-Essen,

Germany.

^3^ Institute for Medical Informatics, Biometry and Epidemiology, University Hospital

Essen, University Duisburg-Essen, Essen, Germany

^4^ Center of Clinical Epidemiology, Institute for Medical Informatics, Biometry and

Epidemiology, Medical Faculty, University Duisburg-Essen, Essen, Germany

^5^ School of Public Health, Department of Epidemiology Boston University, 715 Albany

Street, Talbot Building, Boston, MA 02118, USA

^6^ Institute of Medical Sociology, Medical Faculty, University of Duesseldorf, Duesseldorf,

Germany

*shared senior authorship

Correspondence author:

Dr. Bernd Kowall

Center of Clinical Epidemiology

c/o Institute of Medical Informatics, Biometry and Epidemiology (IMIBE)

Hufelandstraße 55

45147 Essen

Tel: +49-201-92239-295

Fax: +49-201-92239-333

Mail: [bernd.kowall@uk-essen.de](mailto:bernd.kowall@uk-essen.de)

**Appendix 1**: Proposed DAG of the associations between sleep and cognition.


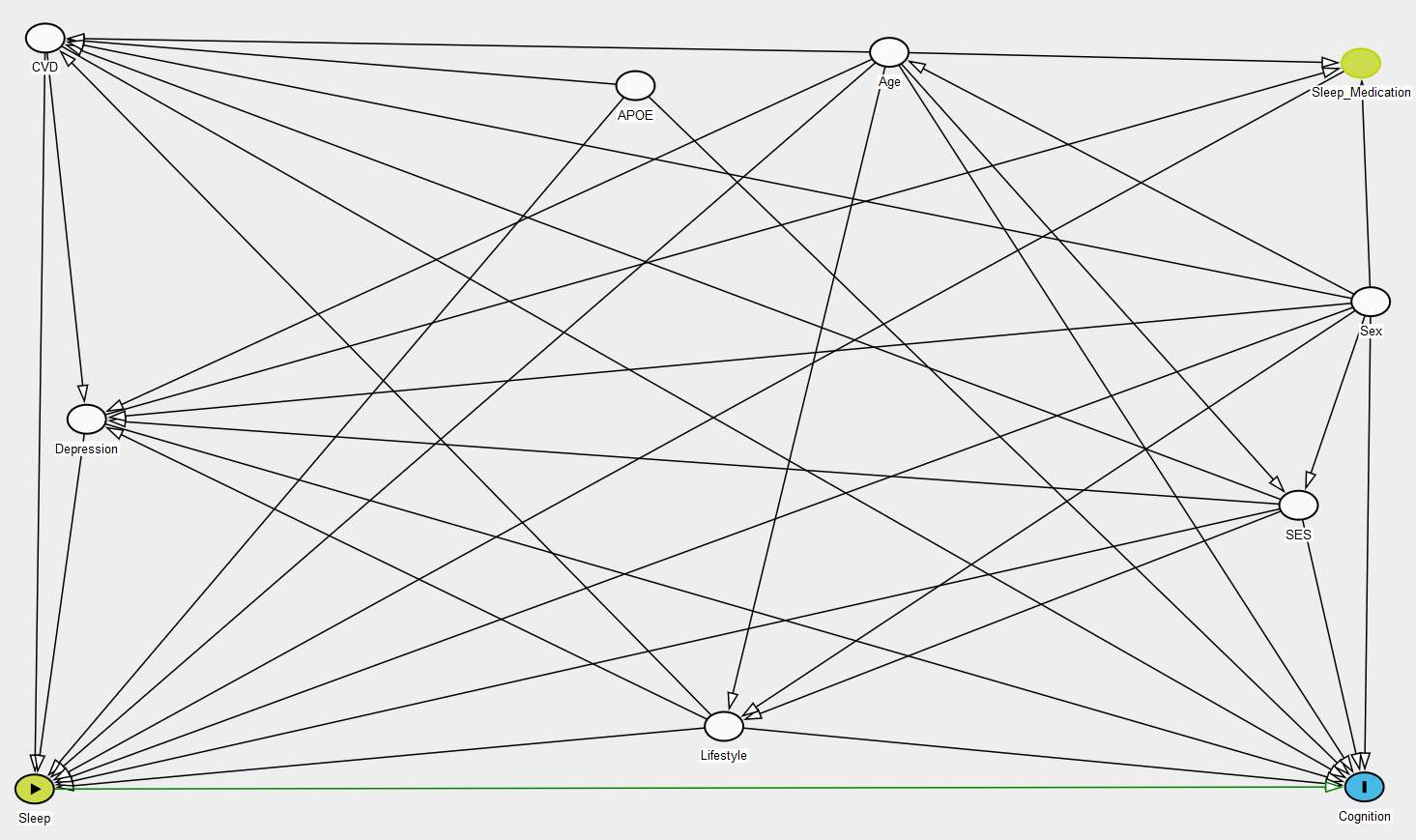


Abbreviations: Abbreviations: CVD: Cardio Vascular Disease; APOE: APOE Genotype: SES: Socio Economic Status

Grey Variables were adjusted for in the final model. Green Variables are an ancestor of the exposure. Green Variable with a play button is the exposure. Blue variable with an I is the outcome.

| **Appendix 2**: Missings in the complete Dataset - the Heinz Nixdorf Recall Study | | |
| --- | --- | --- |
| **Variable** | **n** | **n_Missing_ (Percent)** |
| APOE E4 | 3889 | 925 (19.21%) |
| Age | 4814 | 0 (0.00%) |
| Alcohol | 4691 | 123 (2.56%) |
| PSQI Score | 3741 | 1073 (22.29%) |
| DIS | 4147 | 667 (13.86%) |
| DMS | 4149 | 665 (13.81%) |
| EMA | 4129 | 685 (14.23%) |
| Time in Bed | 3666 | 1148 (23.85%) |
| Time Asleep | 3826 | 988 (20.52%) |
| Total Sleep Duration^1^ | 3822 | 992 (20.61%) |
| Depression Score | 4645 | 169 (3.51%) |
| Framingham Risk Score | 4469 | 345 (7.17%) |
| Height | 4793 | 21 (0.44%) |
| ISCED Years | 4798 | 16 (0.33%) |
| MCI T2 | 2848 | 1966 (40.84%) |
| MET-h/week | 4740 | 74 (1.54%) |
| Sex | 4814 | 0 (0.00%) |
| Smoking Status | 4804 | 10 (0.21%) |
| Waist circumference | 4795 | 19 (0.39%) |
| n_Total_ = 4814 | | |
| Abbreviations: ISCED: International Standard Classification of Education; MET-h: Metabolic Equivalent of Tasks - hours; MCI: Mild Cognitive Impairment; PSQI: Pittsburgh Sleep Quality Index; DIS: Difficulties Initiating Sleep; DMS: Difficulties Maintaining Sleep; EMA: Early-morning Awakening  ^1^ time in bed plus daytime napping. | | |

| **Appendix 3:** Comparison of Adjustment Variables and MCI between Complete Cases and Multiple Imputation | | |
| --- | --- | --- |
|  | Complete Case | Multiple Imputation |
|  | n = 1890 | n = 24800 |
| Sex |  |  |
| Male | 917 (48.5%) | 12070 (48.7%) |
| Female | 973 (51.5%) | 12730 (51.3%) |
| Age |  |  |
|  | 58.0 (7.1) | 57.8 (7.2) |
| Height |  |  |
|  | 169.2 (9.2) | 169.2 (9.2) |
| Waist circumference |  |  |
|  | 92.5 (12.8) | 92.7 (12.9) |
| Smoking Status |  |  |
| Never | 811 (42.9%) | 10720 (43.2%) |
| Current | 705 (37.3%) | 9130 (36.8%) |
| Former | 374 (19.8%) | 4950 (20%) |
| Framingham Risk Score |  |  |
|  | 0.2 (0.2) | 0.2 (0.2) |
| Depression Score |  |  |
|  | 7.2 (5.7) | 7.3 (5.8) |
| ISCED Education Years |  |  |
|  | 14.4 (2.3) | 14.3 (2.4) |
| Met-h |  |  |
|  | 45.3 (44.7) | 44.4 (45.9) |
| Alcohol Consumption |  |  |
|  | 8.8 (15.2) | 8.7 (15.2) |
| APOE E4 Genotype |  |  |
| Non-Carrier | 1423 (75.3%) | 18524 (74.7%) |
| Carrier | 467 (24.7%) | 6276 (25.3%) |
| MCI |  |  |
| No MCI | 1689 (89.4%) | 22110 (89.2%) |
| MCI | 201 (10.6%) | 2690 (10.8%) |

| **Appendix 4:** Comparison of Sleep Variables between Complete Cases and Multiple Imputation | | |
| --- | --- | --- |
|  | Complete Case | Multiple Imputation |
|  | n = 1890 | n = 24800 |
| PSQI |  |  |
|  | 5.5 (3.4) | 5.5 (3.5) |
|  |  |  |
|  | 8.1 (1.2) | 8.1 (1.2) |
| Night Sleep: Recall |  |  |
|  | 7.0 (8.5) | 6.8 (1.1) |
| Total Sleep |  |  |
|  | 8.3 (1.2) | 8.3 (1.2) |
| Difficulties Initiating Sleep |  |  |
| Never | 747 (39.5%) | 9774 (39.4%) |
| Sometimes | 689 (36.5%) | 9040 (36.5%) |
| Often | 316 (16.7%) | 4146 (16.7%) |
| Almost Every Night | 138 (7.3%) | 1840 (7.4%) |
| Difficulties Maintaining Sleep |  |  |
| Never | 195 (10.3%) | 2646 (10.7%) |
| Sometimes | 466 (24.7%) | 6227 (25.1%) |
| Often | 508 (26.9%) | 6565 (26.5%) |
| Almost Every Night | 721 (38.1%) | 9362 (37.8%) |
| Early Morning Awakening |  |  |
| Never | 770 (40.7%) | 10145 (40.9%) |
| Sometimes | 595 (31.5%) | 7682 (31%) |
| Often | 349 (18.5%) | 4582 (18.5%) |
| Almost Every Night | 176 (9.3%) | 2391 (9.6%) |

| **Appendix 5**: Crude and age/sex adjusted Relative Risks for the Association between subjective sleep characteristics and the incidence of mild cognitive impairment at t2: the Heinz Nixdorf Recall Study. | | | | | | | | | | | | | |
| --- | --- | --- | --- | --- | --- | --- | --- | --- | --- | --- | --- | --- | --- |
|  | | | | **Analysis** | | | | | | | | | |
|  |  |  |  | **Complete Case Analysis**  **(N = 1890)** | | | | | **Multiple Imputation**  **(N = 2480)** | | | | |
|  | | **Model:** | | **1** | | | **2** | |  | **1** | | **2** | |
| **Exposure** |  | | **N** | **n (%)** | **RR** | **95% CI** | **RR** | **95% CI** | **n %** | **RR** | **95% CI** | **RR** | **95% CI** |
| Sleep Quality: PSQI Score | ≤ 5 | | 1139 | 105 (9.2%) | 1.00 | Reference | 1.00 | Reference | 9.0% | 1.00 | Reference | 1.00 | Reference |
|  | > 5 | | 745 | 96 (12.9%) | 1.40 | [1.08 to 1.82] | 1.44 | [1.12 to 1.87] | 13.5% | 1.50 | [1.19 to 1.89] | 1.54 | [1.22 to 1.94] |
| Difficulties Initiating Sleep | Never | | 745 | 75 (10.1%) | 1.00 | Reference | 1.00 | Reference | 10.0% | 1.00 | Reference | 1.00 | Reference |
|  | Sometimes | | 688 | 70 (10.2%) | 1.01 | [0.74 to 1.38] | 1.08 | [0.80 to 1.47] | 10.0% | 1.00 | [0.76 to 1.32] | 1.05 | [0.80 to 1.38] |
|  | Often | | 313 | 35 (11.2%) | 1.10 | [0.76 to 1.61] | 1.18 | [0.81 to 1.72] | 12.8% | 1.28 | [0.94 to 1.76] | 1.35 | [0.99 to 1.86] |
|  | Almost every Night | | 138 | 21 (15.2%) | 1.52 | [0.97 to 2.37] | 1.57 | [1.01 to 2.43] | 15.2% | 1.53 | [1.03 to 2.25] | 1.57 | [1.06 to 2.31] |
| Difficulties Maintaining Sleep | Never | | 195 | 20 (10.3%) | 1.00 | Reference | 1.00 | Reference | 10.6% | 1.00 | Reference | 1.00 | Reference |
|  | Sometimes | | 462 | 41 (8.9%) | 0.86 | [0.52 to 1.43] | 0.90 | [0.55 to 1.49] | 9.2% | 0.87 | [0.56 to 1.33] | 0.88 | [0.58 to 1.35] |
|  | Often | | 507 | 50 (9.9%) | 0.96 | [0.59 to 1.57] | 0.98 | [0.61 to 1.59] | 10.2% | 0.96 | [0.64 to 1.46] | 0.96 | [0.64 to 1.45] |
|  | Almost every Night | | 720 | 90 (12.5%) | 1.22 | [0.77 to 1.92] | 1.15 | [0.74 to 1.81] | 12.5% | 1.18 | [0.80 to 1.74] | 1.11 | [0.76 to 1.64] |
| Early-morning Awakening | Never | | 767 | 79 (10.3%) | 1.00 | Reference | 1.00 | Reference | 10.4% | 1.00 | Reference | 1.00 | Reference |
|  | Sometimes | | 592 | 58 (9.8%) | 0.95 | [0.69 to 1.31] | 1.02 | [0.74 to 1.41] | 10.0% | 0.96 | [0.73 to 1.27] | 1.01 | [0.77 to 1.34] |
|  | Often | | 349 | 46 (13.2%) | 1.28 | [0.91 to 1.81] | 1.34 | [0.95 to 1.87] | 13.3% | 1.28 | [0.96 to 1.73] | 1.32 | [0.98 to 1.77] |
|  | Almost every Night | | 176 | 18 (10.2%) | 1.00 | [0.61 to 1.62] | 1.04 | [0.64 to 1.69] | 10.9% | 1.05 | [0.70 to 1.58] | 1.08 | [0.72 to 1.62] |
| Time in Bed | ≤ 5h | | 13 | 3 (23.1%) | 2.72 | [0.97 to 7.67] | 2.98 | [1.17 to 7.55] | 22.8% | 2.53 | [1.13 to 5.66] | 2.80 | [1.27 to 6.15] |
|  | 5 to < 7h | | 209 | 13 (6.2%) | 0.73 | [0.40 to 1.34] | 0.83 | [0.45 to 1.52] | 8.1% | 0.90 | [0.53 to 1.53] | 0.98 | [0.57 to 1.67] |
|  | 7 to < 8h | | 472 | 40 (8.5%) | 1.00 | Reference | 1.00 | Reference | 9.0% | 1.00 | Reference | 1.00 | Reference |
|  | 8 to < 9h | | 655 | 70 (10.7%) | 1.26 | [0.87 to 1.83] | 1.08 | [0.75 to 1.57] | 10.7% | 1.19 | [0.85 to 1.67] | 1.07 | [0.76 to 1.50] |
|  | ≥ 9h | | 535 | 75 (14.0%) | 1.65 | [1.15 to 2.38] | 1.34 | [0.92 to 1.94] | 13.4% | 1.49 | [1.08 to 2.07] | 1.28 | [0.92 to 1.80] |
| Time asleep^1^ | ≤ 5h | | 209 | 28 (13.4%) | 1.24 | [0.82 to 1.86] | 1.31 | [0.88 to 1.95] | 13.5% | 1.20 | [0.83 to 1.73] | 1.26 | [0.88 to 1.82] |
|  | 5 to < 7h | | 611 | 58 (9.5%) | 0.87 | [0.63 to 1.21] | 0.93 | [0.67 to 1.29] | 10.0% | 0.89 | [0.67 to 1.19] | 0.95 | [0.71 to 1.26] |
|  | 7 to < 8h | | 651 | 71 (10.9%) | 1.00 | Reference | 1.00 | Reference | 11.2% | 1.00 | Reference | 1.00 | Reference |
|  | 8 to < 9h | | 351 | 39 (11.1%) | 1.02 | [0.71 to 1.48] | 0.89 | [0.62 to 1.29] | 10.7% | 0.96 | [0.68 to 1.34] | 0.88 | [0.63 to 1.23] |
|  | ≥ 9h | | 62 | 5 (8.1%) | 0.74 | [0.31 to 1.77] | 0.64 | [0.27 to 1.51] | 8.5% | 0.76 | [0.34 to 1.66] | 0.68 | [0.31 to 1.48] |
| Total Sleep Duration^2^ | ≤ 5h | | 7 | 2 (28.6%) | 2.00 | [1.27 to 3.16] | 2.05 | [1.31 to 3.22] | 21.8% | 2.55 | [0.74 to 8.85] | 2.91 | [0.86 to 9.88] |
|  | 5 to < 7h | | 198 | 13 (6.6%) | 1.08 | [0.74 to 1.56] | 1.13 | [0.78 to 1.63] | 8.9% | 1.05 | [0.61 to 1.79] | 1.12 | [0.65 to 1.93] |
|  | 7 to < 8h | | 447 | 33 (7.4%) | 1.00 | Reference | 1.00 | Reference | 8.4% | 1.00 | Reference | 1.00 | Reference |
|  | 8 to < 9h | | 627 | 71 (11.3%) | 1.50 | [1.05 to 2.12] | 1.37 | [0.97 to 1.94] | 11.1% | 1.31 | [0.92 to 1.87] | 1.18 | [0.82 to 1.68] |
|  | ≥ 9h | | 605 | 82 (13.6%) | 1.92 | [1.18 to 3.11] | 1.61 | [0.99 to 2.60] | 13.0% | 1.54 | [1.10 to 2.15] | 1.28 | [0.91 to 1.82] |
| Estimates of relative risk with 95% confidence intervals were obtained from a log-linear model with a Poisson working likelihood and robust standard errors. N(CC) = 1890.  t1: Second visit in the Heinz Nixdorf Recall Study (HNRS); t2: Third visit in the HNRS; CC: complete case; MI: multiple imputation; RR: relative risk; n %: percent of cases; CI: 95% Confidence Interval; PSQI: Pittsburgh Sleep Quality Index; n%: percentage of persons in the category  Model 1 is not adjusted. Model 2 is adjusted for age(cont.) and sex (male/female).  ^1^ Time asleep does not include time awake in bed.  ^2^ Time in bed plus daytime napping. | | | | | | | | | | | | | |

| **Appendix 6:** Relative Risk for the Association Between Subjective Sleep Characteristics and Incident MCI by Age Group: the Heinz Nixdorf Recall Study | | | | | | | | |
| --- | --- | --- | --- | --- | --- | --- | --- | --- |
|  |  | | **Age Group** | | | | | |
|  |  | | **45 to 54** | | **55 to 64** | | **65 to 74** | |
| **Exposure** |  |  | **RR** | **95% CI** | **RR** | **95% CI** | **RR** | **95% CI** |
| PSQI Score |  | ≤ 5 | 1.00 | Reference | 1.00 | Reference | 1.00 | Reference |
|  |  | > 5 | 1.80 | [1.08 to 2.99] | 1.16 | [0.80 to 1.69] | 1.51 | [1.00 to 2.27] |
| Difficulties Initiating Sleep |  | Never | 1.00 | Reference | 1.00 | Reference | 1.00 | Reference |
|  |  | Sometimes | 0.95 | [0.57 to 1.60] | 0.97 | [0.64 to 1.47] | 1.20 | [0.72 to 1.99] |
|  |  | Often | 1.15 | [0.62 to 2.11] | 1.15 | [0.70 to 1.90] | 1.64 | [0.94 to 2.86] |
|  |  | Almost every Night | 1.80 | [0.84 to 3.88] | 0.72 | [0.33 to 1.57] | 2.57 | [1.41 to 4.68] |
| Difficulties Maintaining Sleep |  | Never | 1.00 | Reference | 1.00 | Reference | 1.00 | Reference |
|  |  | Sometimes | 0.93 | [0.43 to 2.01] | 0.85 | [0.45 to 1.59] | 0.91 | [0.40 to 2.08] |
|  |  | Often | 1.02 | [0.47 to 2.22] | 0.73 | [0.38 to 1.38] | 1.06 | [0.51 to 2.21] |
|  |  | Almost every Night | 1.25 | [0.60 to 2.60] | 0.81 | [0.45 to 1.46] | 1.25 | [0.63 to 2.47] |
| Early Morning Awakening |  | Never | 1.00 | Reference | 1.00 | Reference | 1.00 | Reference |
|  |  | Sometimes | 1.03 | [0.61 to 1.75] | 0.91 | [0.59 to 1.40] | 1.13 | [0.69 to 1.86] |
|  |  | Often | 1.26 | [0.69 to 2.28] | 0.89 | [0.54 to 1.49] | 2.00 | [1.25 to 3.21] |
|  |  | Almost every Night | 1.63 | [0.79 to 3.34] | 0.76 | [0.40 to 1.43] | 0.85 | [0.37 to 1.93] |
| Time in Bed |  | ≤ 5h | 1.46 | [0.36 to 5.93] | 5.61 | [1.65 to 19.05] | 4.89 | [1.10 to 21.71] |
|  |  | >5 - <7h | 0.67 | [0.30 to 1.53] | 1.25 | [0.55 to 2.83] | 1.70 | [0.54 to 5.35] |
|  |  | 7 - <8h | 1.00 | Reference | 1.00 | Reference | 1.00 | Reference |
|  |  | 8 - <9h | 0.99 | [0.53 to 1.84] | 1.08 | [0.61 to 1.89] | 1.23 | [0.65 to 2.32] |
|  |  | ≥ 9h | 1.16 | [0.57 to 2.35] | 1.47 | [0.87 to 2.49] | 1.40 | [0.76 to 2.60] |
| Time asleep^1^ |  | ≤ 5h |  |  | 0.60 | [0.29 to 1.26] | 2.30 | [1.29 to 4.08] |
|  |  | >5 - <7h |  |  | 0.92 | [0.59 to 1.41] | 0.95 | [0.53 to 1.70] |
|  |  | 7 - <8h |  |  | 1.00 | Reference | 1.00 | Reference |
|  |  | 8 - <9h |  |  | 0.90 | [0.54 to 1.48] | 0.96 | [0.56 to 1.64] |
|  |  | ≥ 9h |  |  | 0.70 | [0.22 to 2.23] | 0.93 | [0.29 to 2.94] |
| Total Sleep Duration^2^ |  | ≤ 5h | 1.45 | [0.22 to 9.71] | 11.52 | [2.32 to 57.14] |  |  |
|  |  | >5 - <7h | 0.83 | [0.37 to 1.84] | 1.31 | [0.59 to 2.91] | 2.26 | [0.65 to 7.86] |
|  |  | 7 - <8h | 1.00 | Reference | 1.00 | Reference | 1.00 | Reference |
|  |  | 8 - <9h | 1.12 | [0.61 to 2.07] | 1.01 | [0.56 to 1.81] | 1.71 | [0.82 to 3.58] |
|  |  | ≥ 9h | 1.15 | [0.56 to 2.36] | 1.39 | [0.82 to 2.36] | 1.58 | [0.77 to 3.26] |
| Estimates of relative risks with 95% confidence intervals were obtained from a log-linear model with a Poisson working likelihood and robust standard errors. RR: Relative Risk; n %: Percent of Cases; CI: confidence interval. Model is adjusted for sex (male/female), height (cont.), waist (cont.), Framingham CVD risk score (cont.), smoking status (never/former/current), ISC education years (cont.), physical activity (cont.; met-h/week), pure alcohol consumption (cont.; g/week), APOE e4 genotype (carrier/non-carrier), and depression score (cont.). Nocturnal Sleep Duration from the PSQI could not be estimated because not enough incident cases were present in all sleep strata.  ^1^ Time asleep does not include time awake in bed.  ^2^ time in bed plus daytime napping. | | | | | | | | |

| **Appendix 7:** Relative Risk for the Association Between Subjective Sleep Characteristics and Incident MCI by APOE ε4 Carrier Status: the Heinz Nixdorf Recall Study | | | | | |
| --- | --- | --- | --- | --- | --- |
|  | | **APOE ε4 Carrier** | | | |
|  | | **No** | | **Yes** | |
| **Exposure** |  | **RR** | **95% CI** | **RR** | **95% CI** |
| PSQI Score | ≤ 5 | 1.00 | Reference | 1.00 | Reference |
|  | > 5 | 1.61 | [1.28 to 2.02] | 1.21 | [0.82 to 1.76] |
| Difficulties Initiating Sleep | Never | 1.00 | Reference | 1.00 | Reference |
|  | Sometimes | 1.01 | [0.77 to 1.33] | 0.94 | [0.61 to 1.43] |
|  | Often | 1.48 | [1.11 to 1.97] | 1.36 | [0.85 to 2.18] |
|  | Almost every Night | 1.34 | [0.92 to 1.96] | 1.53 | [0.88 to 2.66] |
| Difficulties Maintaining Sleep | Never | 1.00 | Reference | 1.00 | Reference |
|  | Sometimes | 1.13 | [0.69 to 1.86] | 0.70 | [0.39 to 1.25] |
|  | Often | 1.46 | [0.91 to 2.36] | 0.65 | [0.35 to 1.19] |
|  | Almost every Night | 1.66 | [1.06 to 2.62] | 0.77 | [0.45 to 1.32] |
| Early Morning Awakening | Never | 1.00 | Reference | 1.00 | Reference |
|  | Sometimes | 1.02 | [0.77 to 1.36] | 0.80 | [0.52 to 1.23] |
|  | Often | 1.31 | [0.99 to 1.75] | 1.05 | [0.68 to 1.62] |
|  | Almost every Night | 1.58 | [1.14 to 2.19] | 0.78 | [0.45 to 1.36] |
| Time in Bed | ≤ 5h | 1.52 | [1.09 to 2.11] | 1.91 | [1.08 to 3.36] |
|  | >5 - <7h | 1.00 | [0.73 to 1.36] | 1.77 | [1.12 to 2.81] |
|  | 7 - <8h | 1.00 | Reference | 1.00 | Reference |
|  | 8 - <9h | 1.12 | [0.83 to 1.50] | 1.51 | [0.92 to 2.47] |
|  | ≥ 9h | 1.76 | [1.18 to 2.61] | 1.67 | [0.74 to 3.79] |
| Time asleep^1^ | ≤ 5h | 1.14 | [0.81 to 1.62] | 1.44 | [0.86 to 2.41] |
|  | >5 - <7h | 1.09 | [0.82 to 1.45] | 0.87 | [0.55 to 1.36] |
|  | 7 - <8h | 1.00 | Reference | 1.00 | Reference |
|  | 8 - <9h | 0.81 | [0.58 to 1.15] | 1.11 | [0.68 to 1.82] |
|  | ≥ 9h | 0.96 | [0.52 to 1.78] | 0.74 | [0.26 to 2.15] |
| Total Sleep Duration^2^ | ≤ 5h | 1.52 | [1.05 to 2.20] | 2.20 | [1.22 to 3.96] |
|  | >5 - <7h | 1.05 | [0.77 to 1.41] | 1.85 | [1.17 to 2.93] |
|  | 7 - <8h | 1.00 | Reference | 1.00 | Reference |
|  | 8 - <9h | 1.14 | [0.85 to 1.53] | 1.53 | [0.93 to 2.50] |
|  | ≥ 9h | 1.67 | [1.16 to 2.40] | 1.55 | [0.73 to 3.26] |
| Estimates of relative risks with 95% confidence intervals were obtained from a log-linear model with a Poisson working likelihood and robust standard errors. RR: Relative Risk; CI: confidence interval. Model is adjusted for age (cont.) and sex (male/female) height (cont.), waist (cont.), Framingham CVD risk score (cont.), smoking status (never/former/current), ISC education years (cont.), physical activity (cont.; meth/week), pure alcohol consumption (cont.; g/week), and depression score (cont.).  ^1^ Time asleep does not include time awake in bed.  ^2^ time in bed plus daytime napping. | | | | | |
